# Prospective cohort study of the effectiveness of varenicline versus nicotine replacement therapy for smoking cessation in the “real world”

**DOI:** 10.1186/1471-2458-14-1163

**Published:** 2014-11-12

**Authors:** Daniel Kotz, Jamie Brown, Robert West

**Affiliations:** Department of Family Medicine, CAPHRI School for Public Health and Primary Care, Maastricht University Medical Centre, PO Box 616, 6200 MD Maastricht, The Netherlands; Cancer Research UK Health Behaviour Research Centre, University College London, London, WC1E 6BT UK

**Keywords:** Smoking cessation, Varenicline, Nicotine replacement therapy, Behavioural support, Prospective cohort study

## Abstract

**Background:**

It is important to know the comparative effectiveness of varenicline and nicotine replacement therapy (NRT) for smoking cessation when prescribed under routine circumstances and in the general population. Previous estimates relied on cross-sectional data. The objective of the current study was to use longitudinal data to compare the abstinence rates of smokers trying to stop having used varenicline versus NRT on prescription (Rx) when provided with minimal professional support in the general population while adjusting for key potential confounders.

**Methods:**

Prospective cohort study in 270 adults who participated in a household survey, smoked at baseline, responded to the 6-month follow-up survey, and made at least one quit attempt between the two measurements with either varenicline or NRT Rx in their most recent quit attempt. The main outcome measure was self-reported abstinence up to the time of the survey, adjusted for key potential confounders including cigarette dependence (measured at baseline).

**Results:**

Users of varenicline were younger, reported more time spent with urges to smoke at baseline, and were less likely to stop abruptly during their last quit attempt (all p < 0.01). The adjusted odds of abstinence in users of varenicline were 3.83 (95% CI = 1.88-7.77) times higher compared with users of NRT Rx.

**Conclusions:**

Varenicline use with minimal professional support in the general population of smokers appears more effective than NRT Rx in achieving abstinence.

## Background

Results from a network meta-analysis of randomised controlled trials indicate that varenicline, a partial α4β2 receptor agonist, might be more effective than single form nicotine replacement therapy (NRT) in achieving short-term abstinence from smoking [1]. It is important to supplement the evidence from such experimental studies with evidence from observational studies in the ‘real world’ in order to establish generalisability.

Several cohort studies have been conducted which compared varenicline with NRT, and the majority reported a higher effectiveness of varenicline [2–9]. However, these exclusively included clinical samples, e.g. smokers attending stop-smoking services where they receive specialist behavioural support. Most use of varenicline involves a prescription from a clinician with minimal behavioural support and it is important to assess how far the superiority of varenicline extends to this context.

We previously conducted the only study comparing varenicline with NRT when prescribed with minimal professional support in a representative sample of the general population, and showed that varenicline was associated with higher abstinence rates than NRT [10]. However, that study was limited by its cross-sectional design. In order to adjust for confounding, we used a validated measure [11] involving ratings of current urges to smoke assessed at the time of the survey. In smokers who were abstinent at the time of the survey these measures served as a proxy for urges to smoke at the time of the quit attempt, which seemed to be a valid assumption [12]. It would be advantageous to measure smokers’ level of cigarette dependence prior to their quit attempt and follow these smokers up to assess their outcome. We therefore conducted a prospective cohort study in a general population sample comparing the effectiveness of varenicline with NRT on prescription (Rx) when provided with minimal professional support and while adjusting for key potential confounding factors measured at baseline.

## Methods

We used data from the “Smoking Toolkit Study”, which is an ongoing research programme designed to provide information about smoking cessation and factors that promote or inhibit it at a population level [13, 14]. Each month a new sample of approximately 1,800 people aged 16 and over completes a face-to-face computer-assisted survey, of whom approximately 450 are smokers. The methods have been described in full elsewhere and have been shown to result in figures for key variables such as smoking prevalence that are nationally representative [13] (http://www.smokinginengland.info).

### Study population

For the current study, we used aggregated data from respondents to the baseline survey in the period from November 2006 (the start of the survey) to March 2012 (the latest wave of the survey for which 6-month follow-up data were available), who smoked cigarettes (including hand-rolled) or any other tobacco product (e.g., pipe or cigar) daily or occasionally at the time of the survey. These respondents were asked if they were happy to be re-contacted. A follow-up questionnaire was sent to consenting respondents 6 months after baseline. Participants were given £5 ($8) remuneration, and one reminder letter was sent. Of the 27219 smokers at baseline, 5757 (21.2%) were followed up 6 months later. The sample followed up differed from those not followed-up by being more likely to be female, older, less motivated to stop smoking, and reporting higher strengths of urges to smoke at baseline. The differences were small but statistically significant.

Respondents to the 6-month follow-up were asked: “Have you made a serious attempt to stop smoking in the past 12 months? By serious attempt I mean you decided that you would try to make sure you never smoked another cigarette? Please include any attempt that you are currently making.” We only included those respondents who made at least one quit attempt up to 6 months ago.

To identify methods used to stop smoking, respondents were asked “Which, if any, of the following did you try to help you stop smoking during the most recent serious quit attempt?” Respondents could select any of the following: “nicotine replacement product on prescription or given to you by a health professional, Champix (varenicline), attended a stop smoking group, attended one or more stop smoking one-to-one counselling\advice\support session\s, nicotine replacement product (e.g., patches\gum\inhaler) without a prescription.”

We identified 379 respondents who used either varenicline or NRT Rx during their most recent quit attempt. We subsequently excluded respondents who used these medications in combination with stop smoking group or one-to-one counselling or NRT over-the-counter (N = 100), or who had missing data on one or more of the confounding variables (see next paragraph; N = 9). This allowed us to perform a complete case analysis in a sample of 270 smokers at baseline who tried to quit between baseline and 6-month follow-up with either varenicline or NRT Rx, both of which were assumed to being prescribed with brief professional advice.

### Measurements

Our primary outcome was self-reported non-smoking up to the time of the 6-month follow-up survey. Respondents were asked: “How long did your most recent serious quit attempt last before you went back to smoking?”. Those responding “I am still not smoking” were defined as non-smokers. Previous research has shown that self-reported abstinence in surveys of this kind closely reflects true smoking rates and is not subject to the kind of biases observed in clinical trials where there is social pressure to claim abstinence [15, 16].

We measured variables that are potentially associated with the use of smoking cessation treatments and that may also have an effect on abstinence. These potential confounders were chosen a priori. The most important factor was cigarette dependence for which we used two questions measured at baseline. First, time spent with urges to smoke was assessed by asking: “How much of the time have you felt the urge to smoke in the past 24 hours? Not at all (coded 1), a little of the time (2), some of the time (3), a lot of the time (4), almost all of the time (5), all of the time (6)”. Second, strength of urges to smoke was measured by asking “In general, how strong have the urges to smoke been?”: slight (1), moderate (2), strong (3), very strong (4), extremely strong (5). This question was coded “0” for smokers who responded “not at all” to the previous question. Different measures of dependence exist but urges to smoke have been found to be a better predictor of relapse than the more common Fagerström Test for Nicotine Dependence and its components in this particular population [11]. Demographic characteristics we took into account were age, sex, and social grade (AB = managerial and professional occupations, C1 = intermediate occupations, C2 = small employers and own account workers, D = lower supervisory and technical occupations, and E = semi-routine and routine occupations, never workers, and long-term unemployed). With regard to the most recent quit attempt measured at 6-month follow-up, we asked the time since this quit attempt was initiated; the number of quit attempts prior to this attempt that occurred since baseline; and whether respondents cut down first or stopped abruptly without cutting down.

### Data analysis

The simple associations between potential confounders and use of varenicline vs. NRT Rx were assessed with ANOVA for continuous variables and Pearson’s *χ*^2^ for categorical variables.

For the primary analysis, we used a multiple logistic regression model in which we regressed the outcome measure (self-reported non-smoking at 6-month follow-up compared with smoking) on the effect measure (varenicline vs. NRT Rx), adjusted for the above mentioned potential confounders and year of the survey.

## Results

Among the study sample of 270 respondents 193 (71.5%) reported smoking and 77 (28.5%) reported non-smoking at the 6-month follow-up survey. A total of 118 (43.7%) respondents had used varenicline and 152 (56.3%) NRT Rx during their most recent quit attempt. The unadjusted abstinence rates were 39.8% (N = 47) for users of varenicline and 19.7% (N = 30) for users of NRT Rx.

Associations between characteristics of the sample and use of varenicline or NRT Rx are presented in Table 1. Users of varenicline were younger, reported more time spent with urges to smoke at baseline, and were less likely to stop abruptly during their most recent quit attempt at follow-up. Users of varenicline and NRT Rx also differed by social grade, but this difference was non-linear.

**Table 1:** **Associations between sample characteristics and use of varenicline or NRT Rx**

Variable	Varenicline	NRT Rx	P
	(N = 118)	(N = 152)	
Non-smoker at follow-up	39.8 (47)	19.7 (30)	<0.001
Age at baseline, mean (SD)	46.0 (12.2)	51.7 (13.8)	<0.001
Female sex	57.6 (68)	56.6 (86)	0.863
Social grade			
AB	2.5 (3)	10.5 (16)	<0.001
C1	29.7 (35)	17.8 (27)	
C2	23.7 (28)	11.8 (18)	
D	22.0 (26)	18.4 (28)	
E	22.0 (26)	41.4 (63)	
Number of quit attempts prior to the most recent one at follow-up			
0	83.1 (98)	73.0 (111)	0.147
1	12.7 (15)	19.7 (30)	
2	4.2 (5)	7.2 (11)	
Time since last quit attempt started at follow-up			
<=1 week	7.6 (9)	9.2 (14)	0.097
1–4 weeks	8.5 (10)	13.8 (21)	
4–8 weeks	22.0 (26)	18.4 (28)	
8–12 weeks	22.0 (26)	31.6 (48)	
12–26 weeks	39.8 (47)	27.0 (41)	
Stopped abruptly during last quit attempt at follow-up (versus cut down first)	35.6 (42)	52.0 (79)	0.007
Time spent with urges to smoke at baseline, mean (SD)	3.6 (1.3)	3.2 (1.1)	0.006
Strength of urges to smoke at baseline, mean (SD)	2.4 (1.1)	2.3 (1.0)	0.481

Figures are presented as percentage within varenicline/NRT Rx (N), unless stated otherwise. Time spent with urges to smoke: 1 (not at all) to 6 (all the time). Strength of urges to smoke: 0 (no urges) to 5 (extremely strong urges). NRT Rx = nicotine replacement therapy on prescription. Social grade: AB = managerial and professional occupations, C1 = intermediate occupations, C2 = small employers and own account workers, D = lower supervisory and technical occupations, and E = semi-routine and routine occupations, never workers, and long-term unemployed.

The results of our primary analysis are presented in Table 2. The adjusted odds of non-smoking in users of varenicline were 3.83 (95% CI = 1.88-7.77) times higher compared with users of NRT Rx.

**Table 2:** **Unadjusted and adjusted odds of self-reported non-smoking at 6-month follow-up, stratified by use of varenicline or NRT Rx**

	OR (95% CI)	
Smoking cessation medication	Unadjusted OR (95% CI)	Adjusted ^†^ OR (95% CI)
Varenicline (N = 118)	2.70 (1.56-4.64)	3.83 (1.88-7.77)
NRT Rx (reference) (N = 152)	1	1

^†^Odds ratio (OR) adjusted for age, sex, social grade, time since last quit attempt started, number of quit attempts prior to the one in question, stopping abruptly versus cutting down, time spent with urges to smoke, strength of urges to smoke, and year of the survey. 95% CI = 95% confidence interval around OR. NRT Rx = nicotine replacement therapy on prescription.

## Discussion

In this prospective cohort study in a representative sample of smokers from the English general population, use of varenicline during a quit attempt was associated with a higher rate of success of achieving abstinence compared with NRT Rx.

The estimated effect size for the comparison of varenicline versus NRT Rx in our current study (OR = 3.83) was larger than estimated from previous studies, but our sample size was relatively small and the confidence intervals (1.88-7.77) overlap with previous estimates. The largest randomised controlled trial directly comparing varenicline with single form NRT reported an odds ratio of 1.70 at the end of treatment [17], which is close to the lower bound of our the confidence interval. Two cohort studies directly compared varenicline with single form NRT, and reported odds ratios of 1.78 at four weeks after the target quit date [4] and 2.03 at 52 weeks [6].

Our study has several limitations. First, the response to our 6-month follow-up was only 21%, and the response also differed slightly by demographic and smoking characteristics. A higher response would have resulted in increased statistical power, but our sample was large enough to statistically detect the differences in success rates between users of varenicline and NRT Rx. There is no clear mechanism by which non-response bias could have influenced the findings, and the fact that the findings support clinical trial data and data from other real-world settings suggests such bias is unlikely to be a major factor. Second, non-randomised studies are generally vulnerable to confounding. We reduced this risk further than many previous studies by adjusting for tobacco dependence and several other potential confounders. Our rating of urges to smoke is a valid measure of dependence as it predicts success at stopping smoking better than the more common Fagerström Test for Nicotine Dependence in the population we are studying (smokers in England) [11]. However, residual confounding may have occurred as not all factors associated with self-selection of treatment were measured in our survey, such as previous use of NRT or varenicline during a quit attempt, co-morbidity [18] or psychological distress [19]. Third, our self-reported outcome measure of abstinence from smoking was not biochemically validated. In observational studies like ours, however, it is unlikely that misreporting of abstinence is associated with the type of treatment respondents used during the last quit attempt they recall [15, 16]. Fourth, we did not have data on whether NRT users were using one form only or more than one form. The results from two other cohort studies indicate that varenicline may not be substantially more effective than *dual* form NRT [3, 8]. It may be that most of our NRT users were using a single form, and that if they used more than one form the difference from varenicline would be reduced or eliminated. Fifth, most of the respondents followed up had only quit for a few weeks or months. It is possible that the difference between NRT and varenicline is reduced longer term. A further limitation is that we did not have data on actual use of, and adherence to, the medication. However, findings from this same data set have found that NRT Rx increases success rates relative to no use of medication by an amount that is in line with results from clinical trials so it seems unlikely that low NRT adherence in this setting would have made a significant contribution to the difference from varenicline. Finally, we did not have data on the actual behavioural support smokers received. We excluded respondents who used varenicline or NRT in combination with stop smoking group or one-to-one counselling. Hence, we found it reasonable to assume that these medications were prescribed with minimal professional support. Nevertheless, there still might be slight variations in the minimal professional support smokers received. If, hypothetically, such minimal support would be systematically “better” in users of varenicline (e.g., in terms of instructions on how to use the medication), this would overestimate the effectiveness of this medication compared with NRT.

As far as we are aware our study is the first prospective cohort study in a general population sample directly comparing the effectiveness of varenicline with NRT Rx when provided with minimal professional support. Our study included all smokers aged 16 years or older who made a quit attempt, including those who smoke less than 10 cigarettes per day - a subgroup that constitutes one third of current smokers in England [20] and is usually excluded from clinical trials.

## Conclusion

In conclusion, this longitudinal study provided further support for a benefit of varenicline compared with NRT Rx as used by the general population of smokers with limited behavioural support. Future research should address the comparative effectiveness in the long term.

### Ethical approval

The study was granted ethical approval by the University College London ethics committee.
